# Supplementary material for: Experimentally Tuned Protein-RNA Rosetta Score Function using Bayesian Optimization
Source: bioRxiv. 2026 Jul 20:2026.07.17.739244. Preprint. [Version 1] doi: 10.64898/2026.07.17.739244 (PMC13419731; doi:10.64898/2026.07.17.739244)
Supplement: Supplementary file 1 [file media-1.pdf]

Supplementary Information

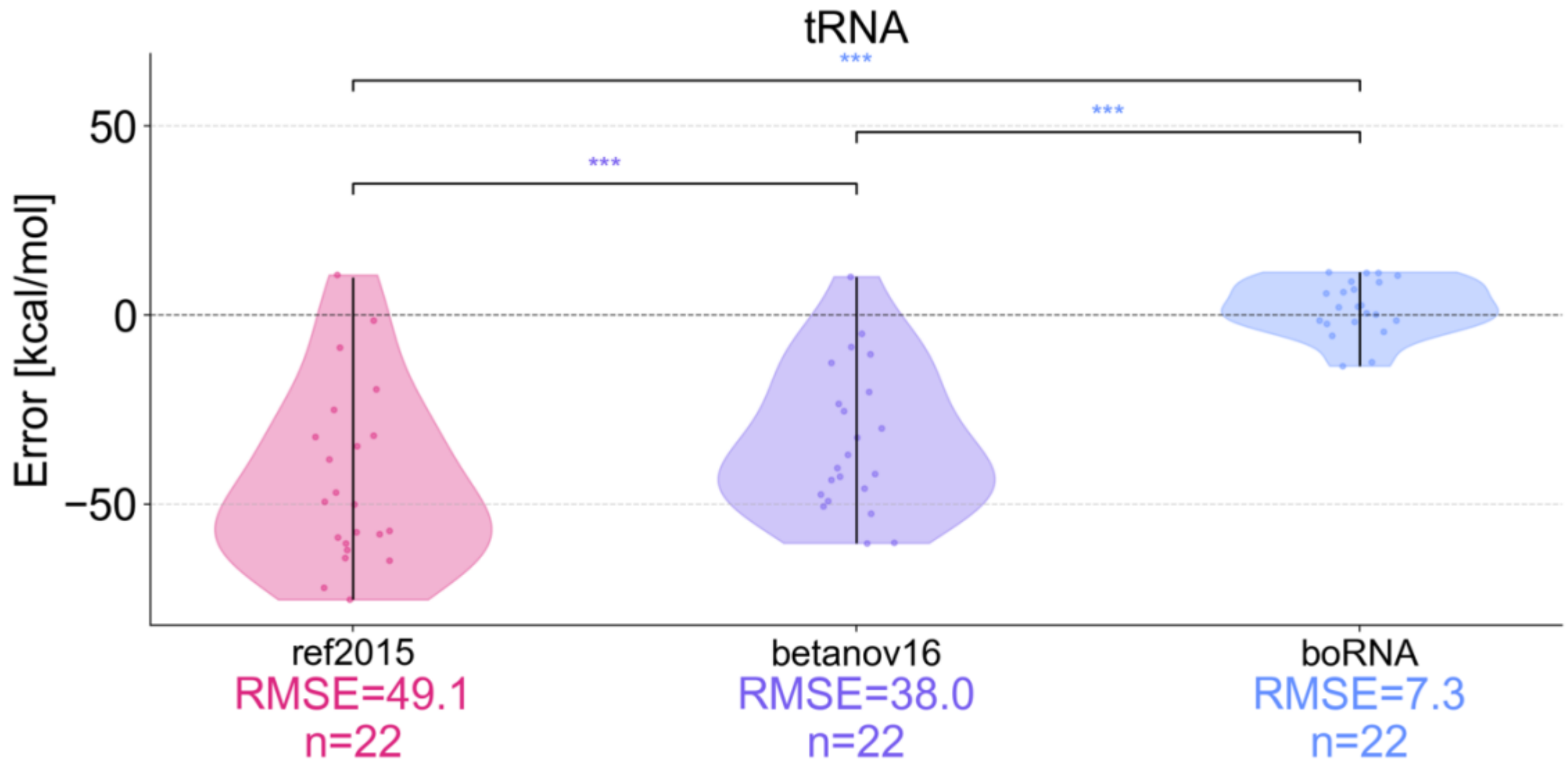

Supplementary Figure 1. tRNA Prediction Error.

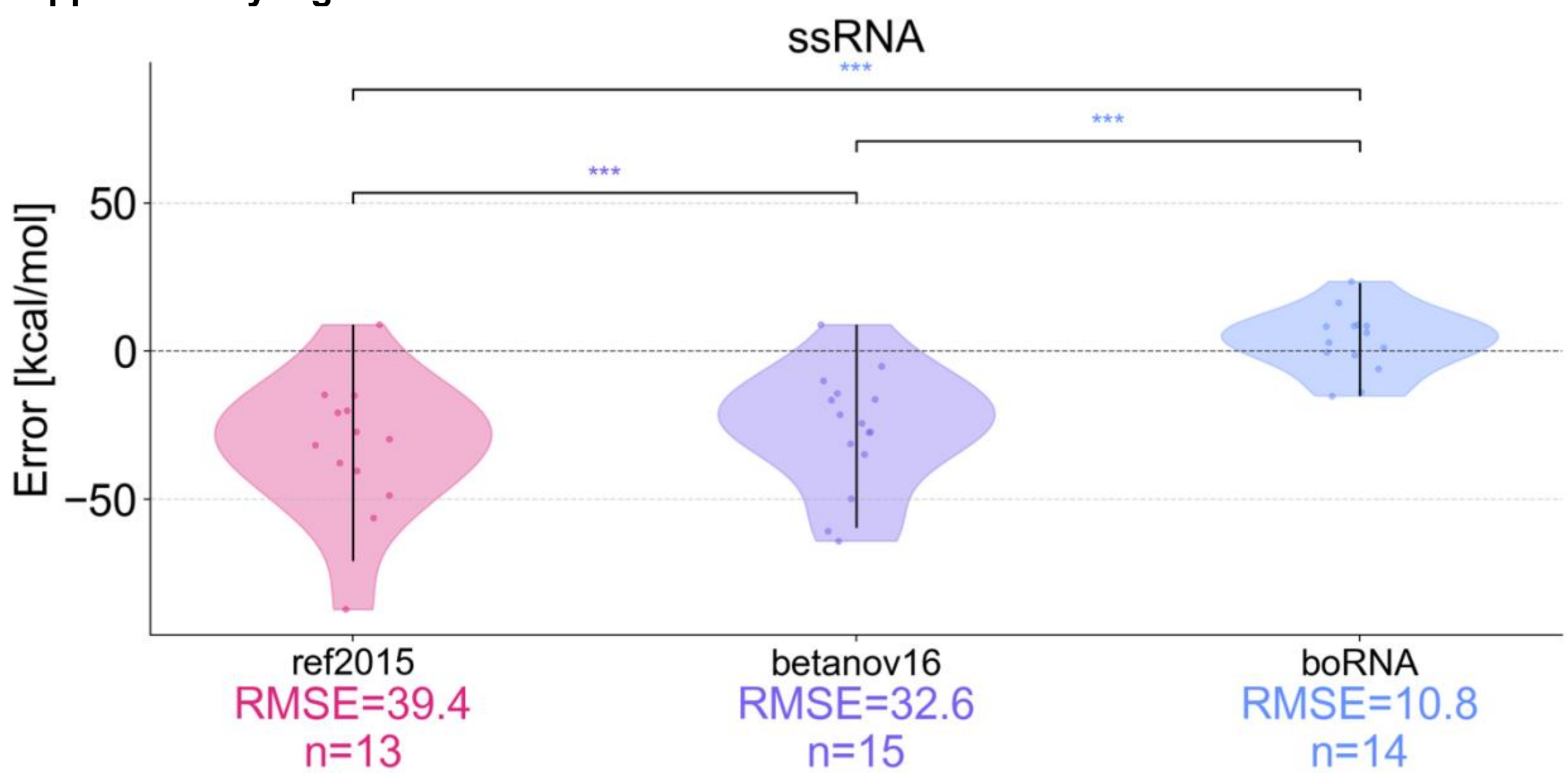

Supplementary Figure 2. ssRNA Prediction Error.

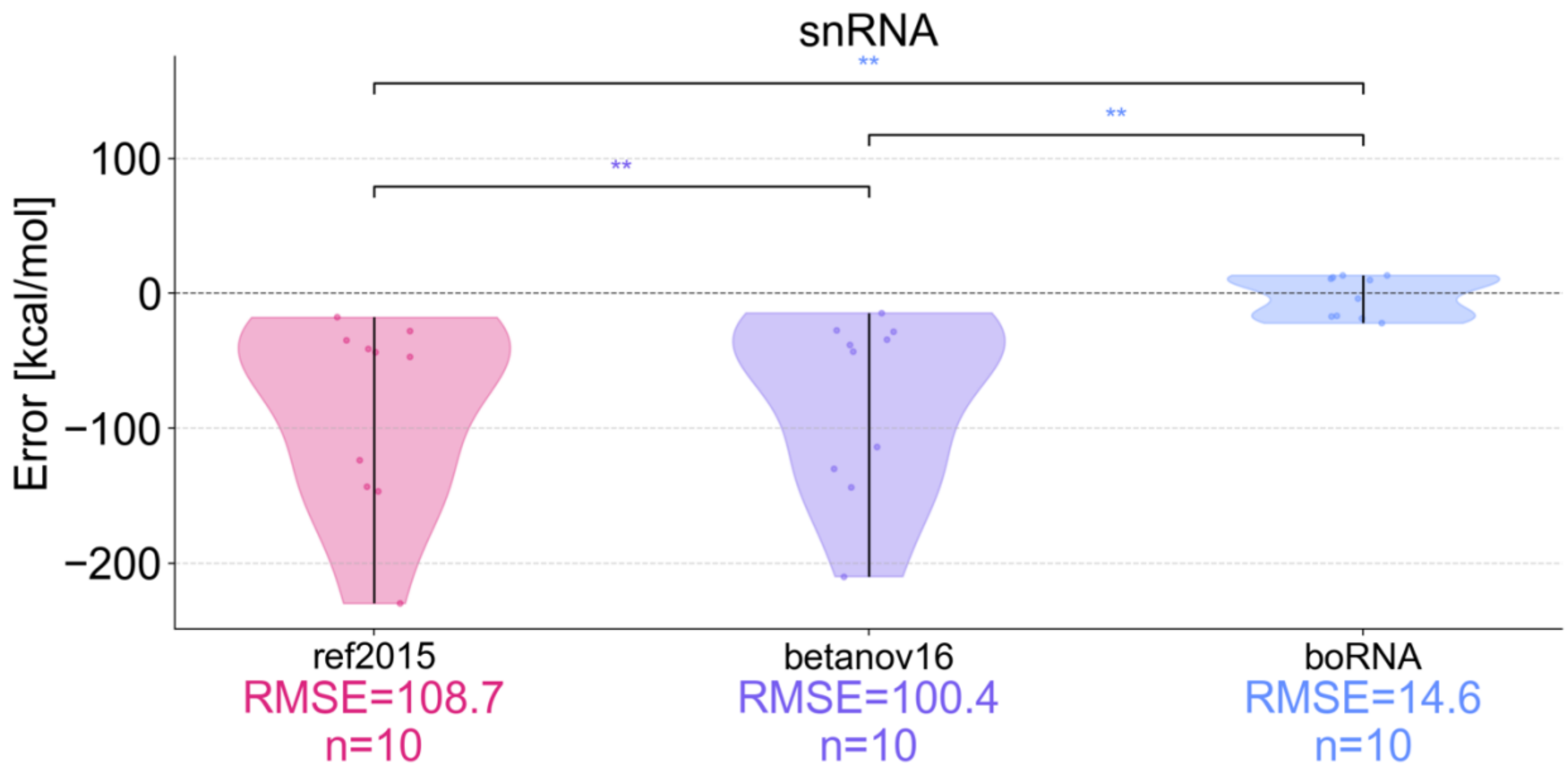

Supplementary Figure 3. snRNA Prediction Error.

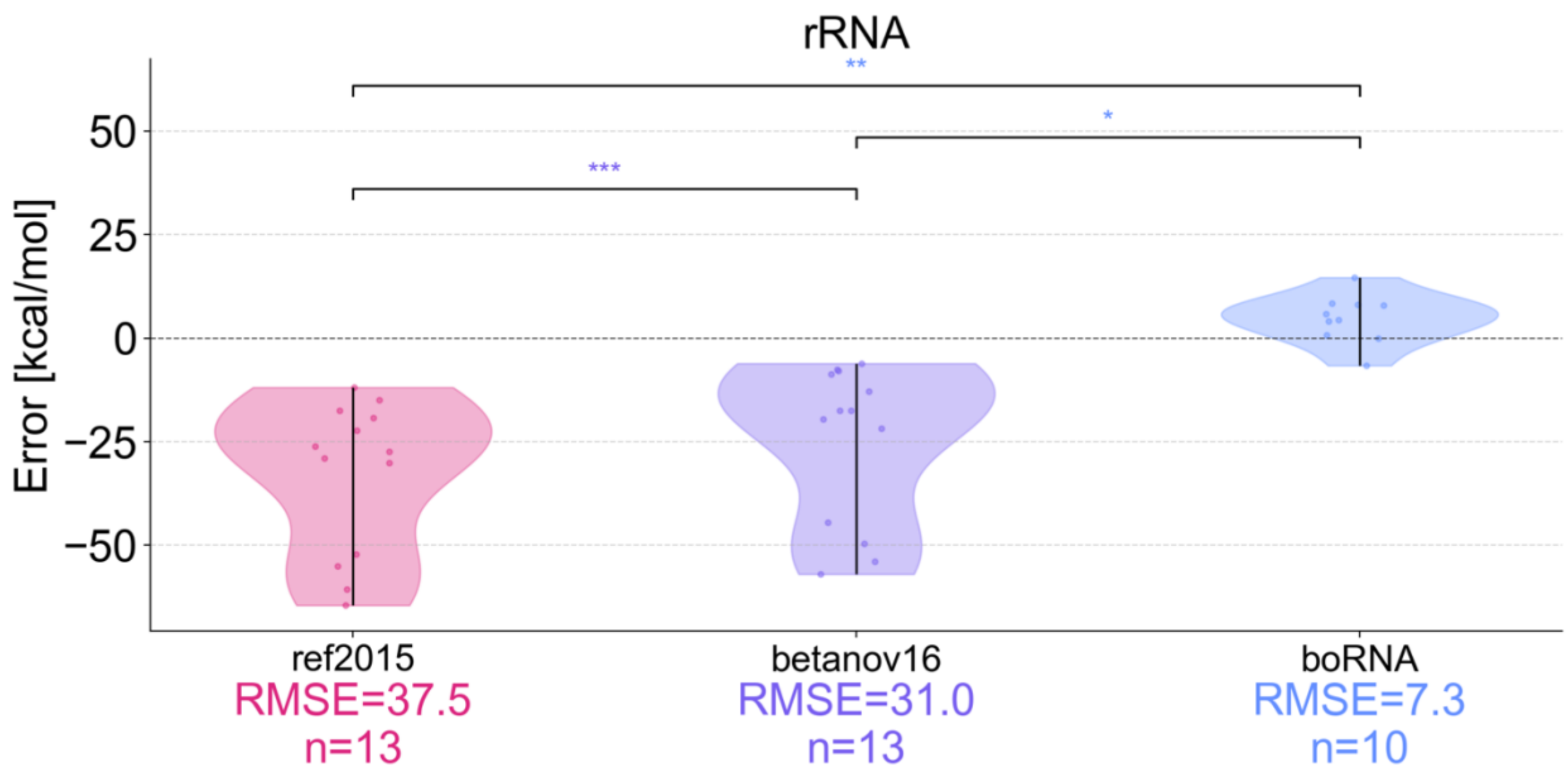

Supplementary Figure 4. rRNA Prediction Error.

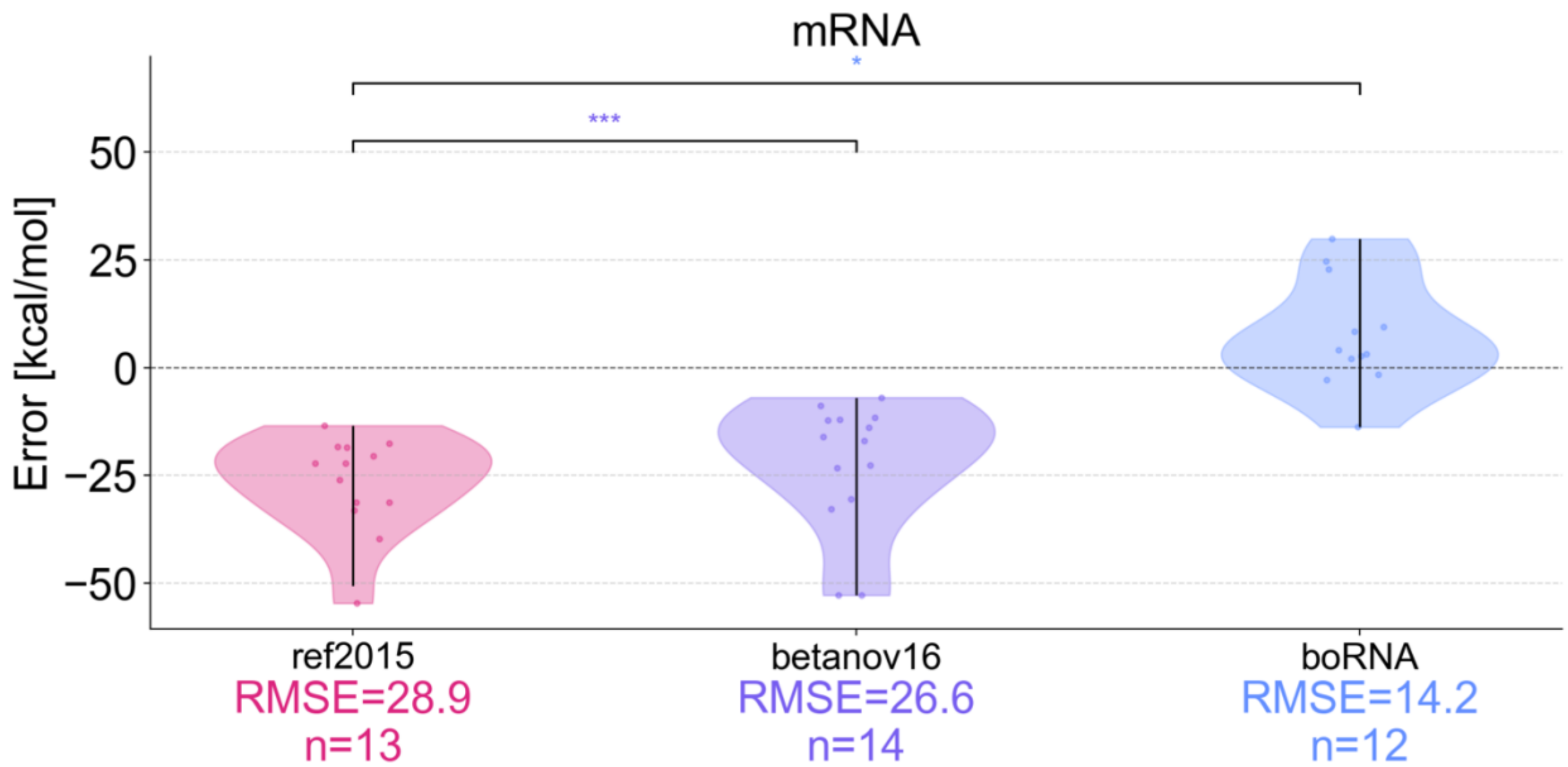

Supplementary Figure 5. mRNA Prediction Error.

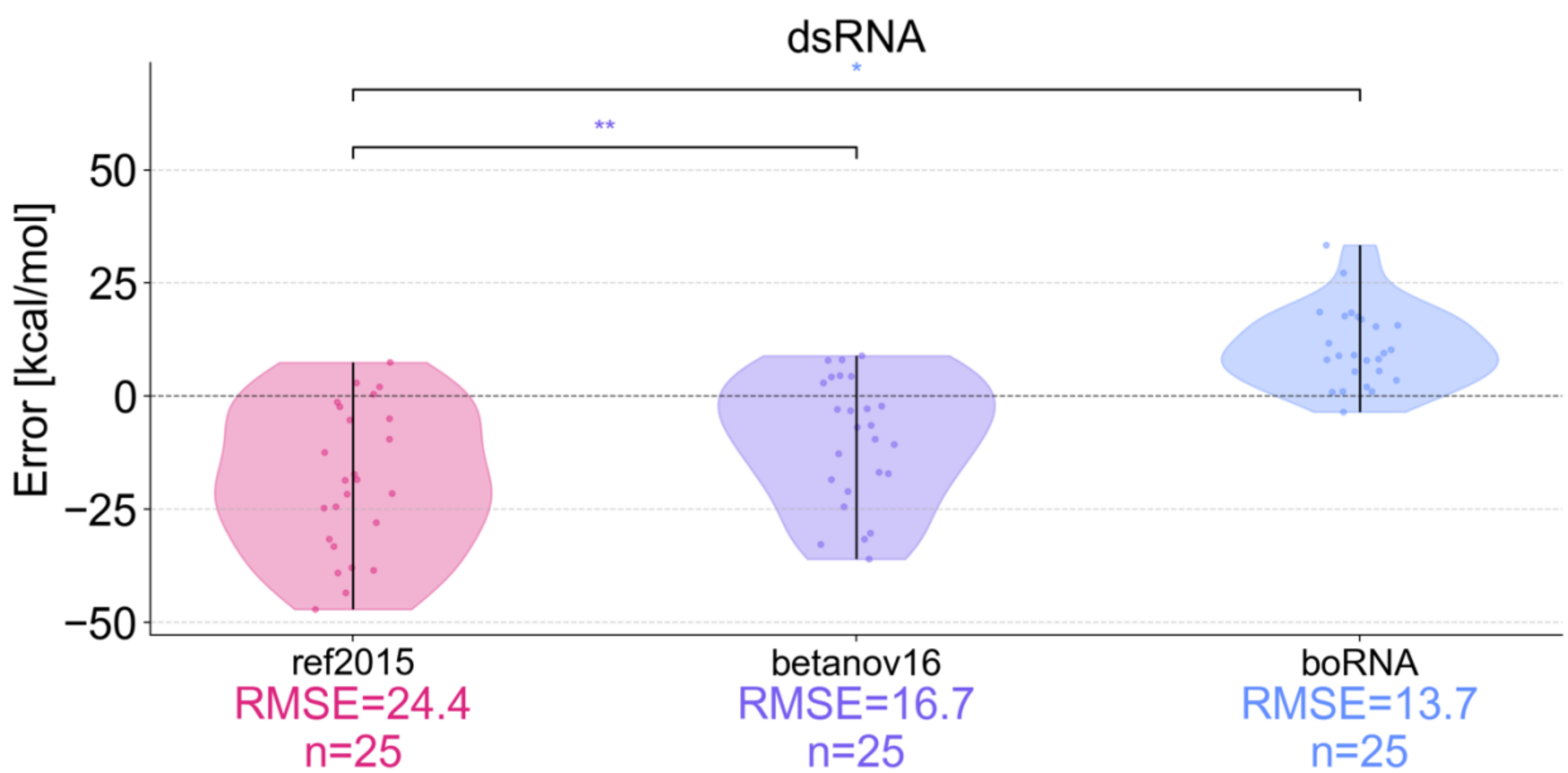

Supplementary Figure 6. dsRNA Prediction Error.

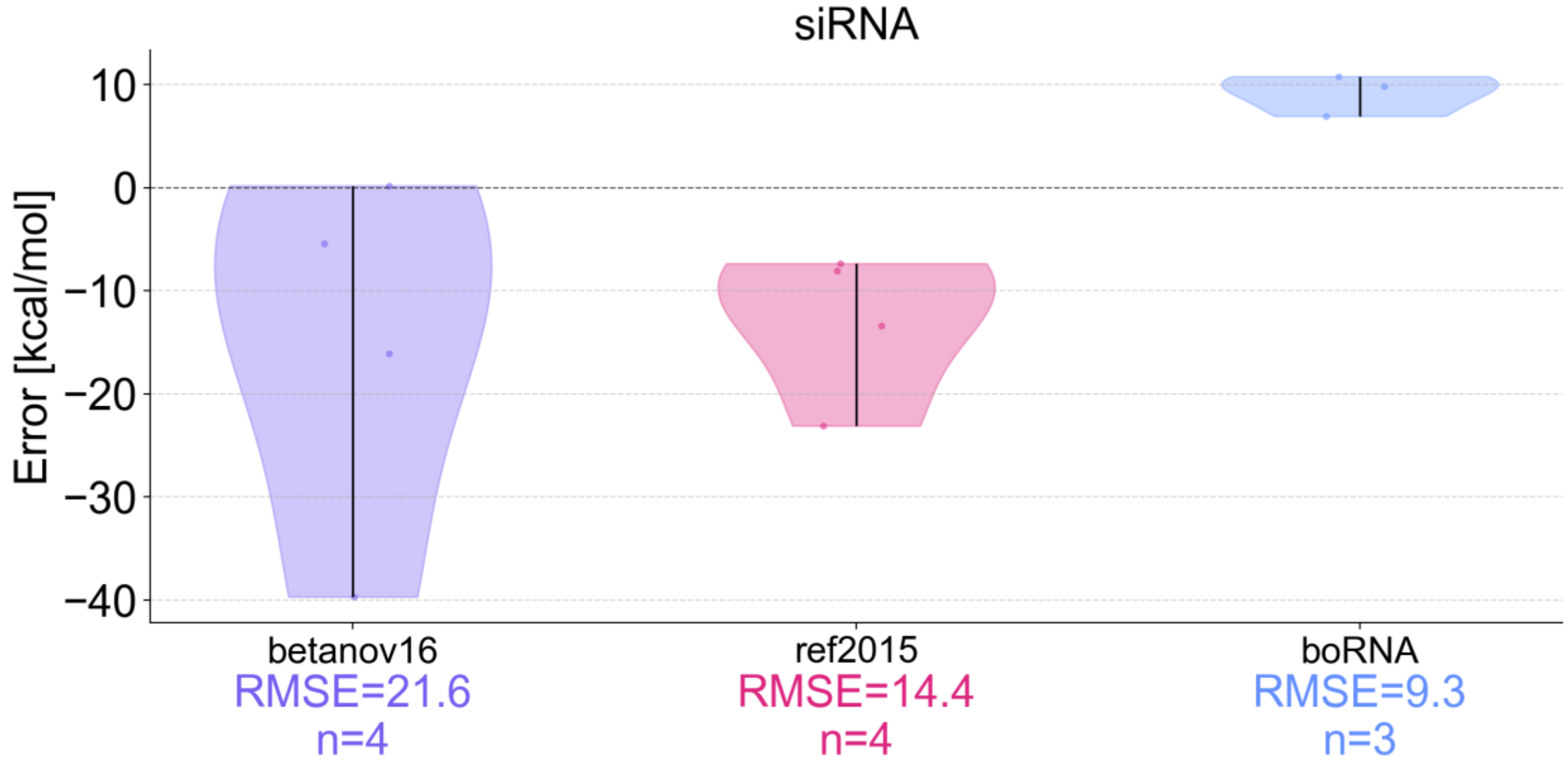

Supplementary Figure 7. siRNA Prediction Error.
